# Temporal Dynamics of EEG Reflect Continuous Error Correction during Force Control

**DOI:** 10.1523/JNEUROSCI.1513-25.2026

**Published:** 2026-07-06

**Authors:** Nicholas Menghi, Elio Balestrieri, Damiano Grignolio, Giorgio Coricelli, Clayton Hickey

**Affiliations:** ^1^Max Planck for Human Cognitive and Brain Sciences, Department of Psychology, Leipzig 04103, Germany; ^2^Center for Information Technology, University of Münster, Muenster 48149, Germany; ^3^Center for Mind/Brain Sciences (CIMeC), University of Trento, Rovereto 36068, Italy; ^4^Department of Economics, University of Southern California, Los Angeles, California 90007; ^5^Laboratory for the Psychology of Child Development (LaPsyDÉ), UMR CNRS 8240, Paris 75005, France; ^6^Centre for Human Brain Health and School of Psychology, University of Birmingham, Birmingham B15 2TT, United Kingdom

**Keywords:** EEG, feedback loop, force control, motor control, sensory feedback

## Abstract

Effective motor control depends on the ability to monitor performance and make continuous corrections. While many studies focus on discrete errors, everyday actions often require ongoing feedback-based adjustments. Here, we used an isometric force control task with electroencephalography (EEG) to investigate the neural dynamics supporting real-time error correction. Participants (13 females, 10 males) maintained a constant grip force with or without continuous visual feedback. With feedback, behavior showed ∼6 Hz rhythmic fluctuations, consistent with active correction. These fluctuations were statistically linked to EEG activity across theta, beta, and alpha bands—oscillations linked to performance monitoring, updating, and attentional control. Without feedback, performance decayed linearly, and the corresponding neural signatures were reduced. These findings suggest that continuous sensory feedback engages a dynamic feedback loop involving distinct neural processes that support adaptive behavior. Our results highlight the importance of oscillatory activity in tracking and correcting moment-to-moment fluctuations in force, offering insight into the neural basis of feedback-loop force control.

## Significance Statement

Most research on error correction has focused on discrete actions, yet everyday behaviors require continuous, fine-grained adjustments. In this study, we show that when people use visual feedback to maintain a stable force output, their behavior fluctuates rhythmically ∼6 Hz, a signature mirrored in brain oscillations across theta, beta, and alpha bands. These patterns reflect a temporal cascade of neural processes that monitor performance and initiate correction. In the absence of visual feedback, performance decays. Our findings reveal how continuous sensory feedback drives a dynamic neural feedback loop that supports real-time motor control. This work advances our understanding of how the brain sustains goal-directed action and highlights the functional role of oscillations in guiding adaptive behavior during continuous tasks.

## Introduction

Motor control and error correction are processes that enable humans to produce precise and adaptive actions. Successful performance depends on continuous monitoring and correction of motor output. When reliable sensory feedback is available, deviations can be rapidly detected and corrected; when it is absent, performance tends to drift. Sensory feedback provides continuous updates about actions and accuracy (Shadmehr et al., 2010; Ullsperger et al., 2014). When external feedback is unavailable, regulation depends on internal representations.

In the lab, studies of feedback in force control have primarily focused on vision, typically using isometric grip-force tasks in which participants maintain a target force (Vaillancourt and Russell, 2002; Baweja et al., 2010; Poon et al., 2012; Abolins and Latash, 2022; Menghi et al., 2025). When visual feedback is provided, participants can adapt their grip force to approximate the target. In its absence, the internal representation degrades due to noise (Slifkin and Newell, 1999; Manohar et al., 2015), limited memory capacity (Vaillancourt and Russell, 2002; Poon et al., 2012), and absence of error signals (Baweja et al., 2010; Abolins and Latash, 2022). Although proprioceptive feedback is continuously available and contributes to force control (Scheidt et al., 2005, 2010), it alone does not reliably support sustained performance (Menghi et al., 2025).

The feedback loop between sensory input, performance monitoring, and motor adjustment relies on coordinated neural processes. Interactions between somatosensory and motor cortices update internal models of action (Monaco et al., 2006; Noble et al., 2013; Sartin et al., 2023), forming a closed-loop system that compares expected and actual outcomes online (Li et al., 2023). These dynamics are reflected in oscillatory brain activity, particularly in theta (4–8 Hz) and beta (12–30 Hz) bands, which appear to orchestrate the continuous evaluation and adjustment of motor output. Brain activity in these frequency ranges has been closely associated with the coordination of performance monitoring (Kilavik et al., 2013), motor inhibition (Logan and Cowan, 1984; Hannah and Aron, 2021; Wessel and Anderson, 2023), and adaptive responses to errors (Little et al., 2019).

Theta oscillations (∼6 Hz), originating from the medial frontal cortex and supplementary motor areas, support conflict detection and performance monitoring (Nachev et al., 2008; Cavanagh and Frank, 2014; Cohen, 2014). Theta is involved in error detection and in the orchestration of performance adjustments, creating a temporal reference that guides information processing and the optimization of goal-directed behavior. When an error is detected, beta oscillations, associated with maintaining ongoing motor states (Baker, 2007; Engel and Fries, 2010), are reduced, enabling corrective responses (Neuper and Pfurtscheller, 2001; Chakarov et al., 2009; Huster et al., 2013; Duque et al., 2017). Alpha oscillations support attentional reallocation during feedback-based correction (Carp and Compton, 2009; Klimesch, 2012; Savoie et al., 2018; Wilken et al., 2023).

Prior studies have focused on neural responses to discrete, one-shot errors (Holroyd and Coles, 2002; Yeung et al., 2004; Pezzetta et al., 2018). However, real-world behavior involves continuous discrepancies requiring sustained monitoring, and the mechanisms supporting such regulation remain less understood (Cohen, 2016; Wilken et al., 2023).

To address this gap, we combined an isometric force control task with electroencephalography (EEG) to investigate the neural mechanisms supporting online motor correction. Participants maintained a constant grip force at a target level with and without continuous visual feedback. This allowed us to track rhythmic fluctuations in behavioral adjustment. We aimed to capture the corresponding dynamic changes in neural activity, offering a window into the closed-loop system of motor control.

By examining the temporal relationship between force deviations and EEG signals, we sought to characterize the cortical processes that support online error detection and correction. Our results show that when visual feedback was available, force output exhibited rhythmic fluctuations at ∼6 Hz, consistent with online correction. These behavioral oscillations were associated with a pattern of EEG activity in the theta, beta, and alpha bands, which tracked performance and predicted adjustments. In contrast, when feedback was absent, performance decayed, and neural responses shifted, reflecting reduced engagement of online correction.

## Materials and Methods

### Participants

Twenty-three participants (13 females, 10 males; mean age 24.2 years; range 20–28 years) gave informed consent before completing the experiment. The participants were all right-handed and naive to the purpose of the experiment. Two female participants were excluded from the analysis of the experiment because they commonly failed to respond, particularly in experimental conditions where feedback was not provided, resulting in a force error that was >3 standard deviations from the group mean. The experiment took ∼2 h to complete, and all participants received a 20 euro reimbursement. All gave informed written consent, and the study procedure was approved by the local institutional review board of the University of Trento.

### Apparatus and stimuli

Participants sat at ∼60 cm from a computer monitor (VIEWPixx/EEG 22ʺ; 1,920 × 1,080; 100 Hz) in a dimly illuminated room with their right hand lying over the table grasping a hand dynamometer. The dynamometer (HD-BTA Vernier) was used to record power grip force effort in Newtons (N) with an accuracy of ±0.6 N. This dynamometer is a strain gauge-based isometric force sensor which amplifies force and converts it into a voltage signal. The voltage signal was transferred to an Arduino Uno through Vernier interface shield hardware and subsequently to an acquisition computer. The force signal was sampled at 100 Hz. During the experiments, signals from this sensor were sent to MATLAB (The MathWorks) for visual real-time feedback of participant’s effort exertion. Feedback was updated at a frequency rate of 100 Hz.

**Figure 1.**
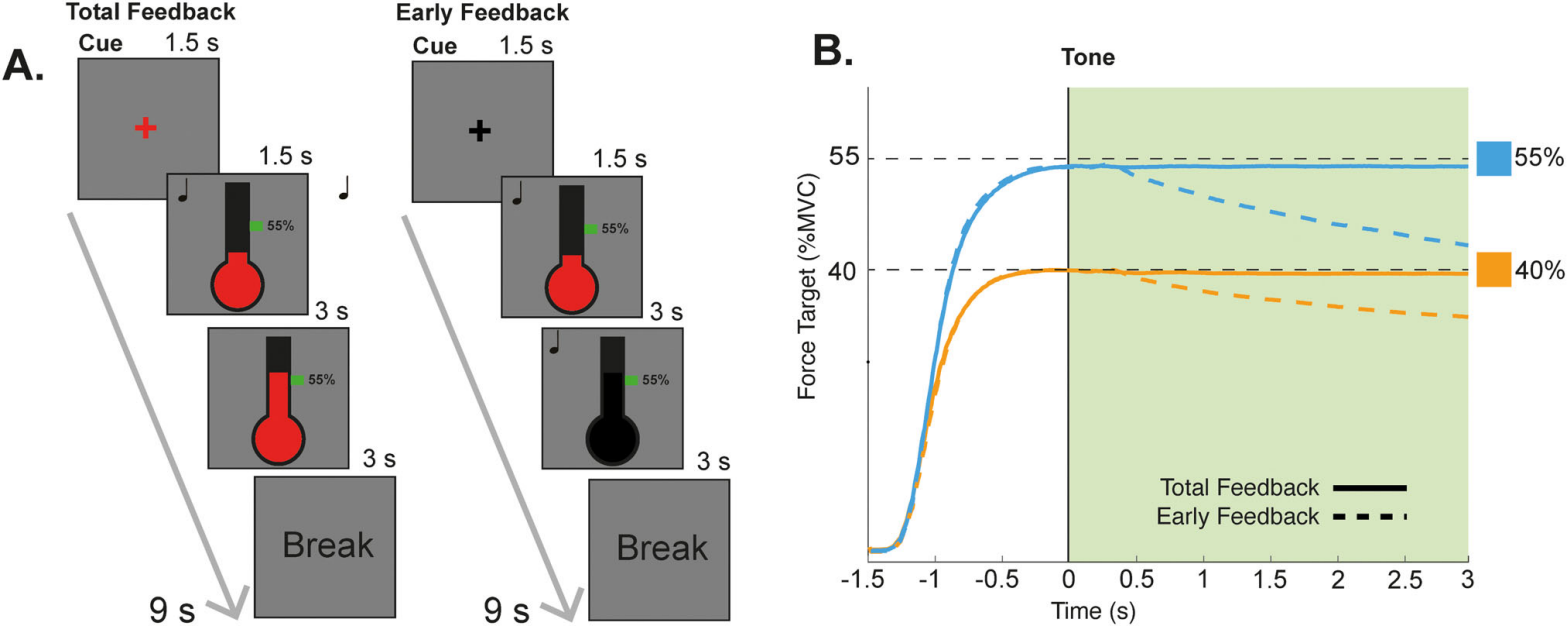
***A***, Task schematics. Each trial began with the presentation of a fixation cross (1.5 s), which signaled the upcoming condition through its color (red or blue). An auditory cue then marked the start of the trial, triggering the appearance of the feedback display. If present, feedback was shown as a red fluid inside a stylized thermometer. The task lasted 4.5 s, consisting of a 1.5 s force estimation phase and a 3 s maintenance phase, each indicated by an auditory cue. This was followed by a 3 s relaxation period, making each trial 9 s in total. Participants received feedback throughout both force estimation and maintenance phases in the Total Feedback condition, while in the Early Feedback condition, feedback was provided only during the force estimation phase. At the end of each block, a message prompted participants to relax. ***B***, Force estimation and maintenance. This panel shows the average performance across participants in the total feedback and early feedback conditions and in the two different force targets.

Presentation of visual stimuli and acquisition of behavioral data was accomplished using PsychToolBox (Brainard, 1997) and custom MATLAB scripts. Before beginning the experiment, participants were requested to exert the most force they could on the dynamometer three times, each time for 3 s., with 10 s of rest between each instance. The maximal voluntary contraction (MVC) was computed as the average of the highest peaks achieved in each of these trials. The trial sequence is illustrated in Figure 1*A*. Each experimental trial began with a cue indicating the feedback condition, and then a target force appeared, which was randomly selected from two possibilities and calculated as a percentage of MVC (40 and 55%). Participants attempted to match this target force level with the hand dynamometer using a whole-hand power grip. For half of the trials, participants were presented with online visual feedback; for the other half, they were presented with visual feedback for 1.5 s only; then they had to rely on somatosensory inputs only. The early feedback condition was included as a control condition to compare how the system initially fails to recalibrate once that feedback is removed, providing insight into error recalibration versus performance decay.

When visual feedback was present, it took the form of a stylized black thermometer that was displayed at the center of an otherwise uniform dark gray background. The thermometer became increasingly red as force was exerted on the dynamometer. A green square on the thermometer indicated the target force output. When visual feedback was absent, the thermometer stayed on screen but the red “fluid” was not presented. Task performance lasted 4.5 s and began with an auditory tone indicating the beginning of a 1.5 s force estimation period, during which participants were to adjust the force to the target value. A tone subsequently indicated the beginning of a 3 s maintenance period, and a final tone indicated the end of the trial. The experiment was composed of 15 practice trials followed by 160 experimental trials divided into eight blocks, with breaks between blocks.

### EEG preprocessing

EEG was recorded at 1 kHz from 64 Ag/AgCl electrodes mounted in an elastic cap (10/20 montage) using the BrainAmp-DC system and Brain Vision Recorder software (Brain Products). Additional electrodes were placed at the left and right mastoids and 1 cm lateral to the outer canthus of the left eye. All electrodes were referenced during the recording to the right mastoid. Electrode impedances were kept below 10 kΩ. Preprocessing was carried out using the FieldTrip Toolbox for MATLAB (Oostenveld et al., 2011).

Continuous data were rereferenced to the common average and high-pass filtered at 0.1 Hz with a zero-phase sixth–order Butterworth filter. The data were epoched from 2.5 s before the beginning of the maintenance period (Fig. 1*B*) to 3.5 s following it. We visually inspected these epochs to remove trials containing muscle activity and electrical artifacts and to identify bad channels, which were interpolated to the weighted average of the neighboring electrodes. Rejected trials were excluded from all further analyses.

Fast independent component analysis (fastICA; Hyvärinen, 1999) was then computed from the epoched data. ICA components representing eyeblinks, eye movements, and sustained high-frequency noise were visually identified and removed from the data. EEG epochs were low-pass filtered with a cutoff of 100 Hz and notch filtered at 50 Hz. Finally, we visually reinspected the epochs to ensure no residual artifacts.

### Dynamometer spectral analysis

Our main goal was to evaluate the presence of periodic phenomena during the maintenance phase and to characterize these phenomena for their peak frequency and range.

We thus first applied a series of preprocessing steps to the signal recorded from the dynamometer over time. We first selected the 3 s corresponding to the maintenance phase. For each participant and each trial, the signal was first normalized in the percentage of the MVC, detrended (first order) and *z*-scored along the time dimension.

**Figure 2.**
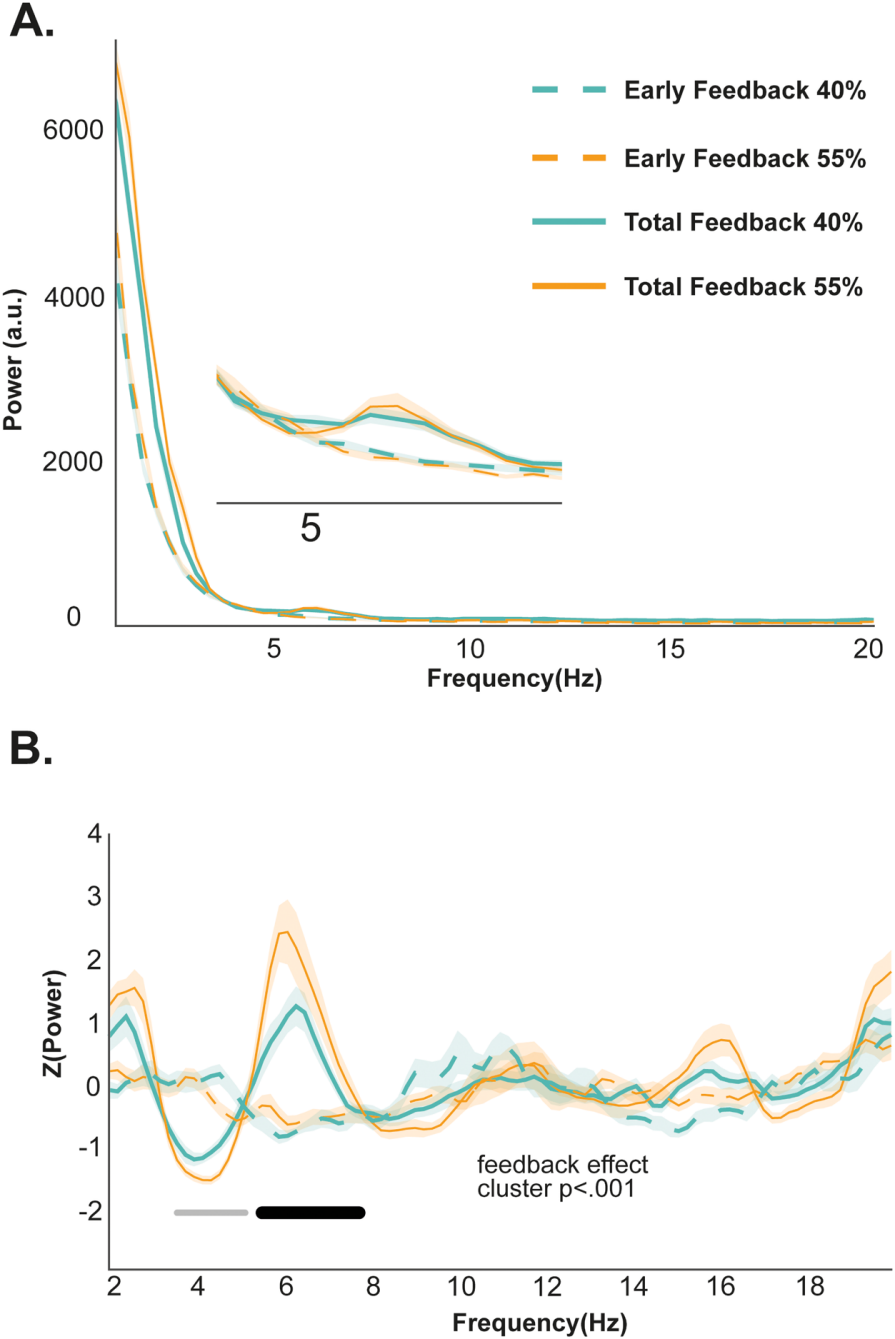
Spectral and linear analyses of force output across feedback conditions. ***A***, Power spectral density of the force signal across the different feedback and target conditions. Feedback condition shows a 6 Hz rhythmic fluctuation during the exertion. This peak is absent in the no-feedback condition. This is more evident in the subpanel, which reflects a magnification of the 4–8 Hz portion of the larger panel. ***B***, Spectral density after removing the fractal 1/*f* component from the signal. In both panels, the shaded area represents standard error of the mean (SEM).

As illustrated in Figure 2*A*, the dynamometer signal is characterized by a strong fractal (aperiodic) component: signal power at low frequencies is large and varies as a nonlinear function of frequency. The results demonstrate conditional differences in this pattern, and this makes it difficult to ascribe effects at specific frequency spectra to changes in brain oscillations. To separate this fractal effect from periodic effects in the dynamometer data and to facilitate estimation of peak frequency and the range of frequency effects, we separated the fractal from the oscillatory component using the IRASA algorithm (Wen and Liu, 2016) as implemented in FieldTrip (Oostenveld et al., 2011). The periodic component was obtained by dividing the spectra by the fractal component, yielding, for each participant, one spectral profile of the oscillatory component contained in the dynamometer signal in each combination of force (40 vs 55%) and feedback (present vs absent).

One goal of the study was to identify oscillatory phenomena that varied as a function of feedback. For this reason, in each frequency bin from 2 to 20 Hz, we computed a linear mixed effect model (LMEM; implemented in Python statsmodels 0.14.4) with force and feedback as main effects and subjects as a grouping variable to account for repeated measures as follows:

power∼feedback+force+(1|subj).

The density of frequency bins and the extension of the window of interest in the frequency domain implied a high number of LMEM models tested, which made it necessary to account for multiple comparisons. This was achieved by applying a cluster permutation approach (Maris and Oostenveld, 2007), where we first defined clusters by selecting the contiguous, absolute *z* values exceeding the critical threshold of the 95th percentile of a normal distribution. All the contiguous values in each cluster were then summed together. Surrogate null datasets were created by shuffling experimental labels within each participant 1,500 times and recalculating the model above. We enforced a stratified permutation scheme, where the experimental labels were swapped either for the feedback condition or for the force condition, avoiding in this way to introduce spurious interaction effects in the surrogate dataset that could have hampered the reliability of the permutation statistics. In each of the shuffled datasets, we repeated the computation of the LMEM in each frequency bin, as well as the clustering, and in each permutation, we collected the cluster statistic with the highest absolute value into a distribution of cluster statistics under the null hypothesis. Finally, we compared the cluster statistics obtained by our tests on the empirical data to the distribution of cluster statistics under the null hypothesis, and we rejected the null hypothesis if a cluster statistic was below the 2.5th or above the 97.5th percentiles of the permutations.

### EEG–dynamometer analysis

To identify alignment between behavioral and EEG datasets, we downsampled the EEG data to 100 Hz to match the sample rate of the behavioral data and focused on results observed in both signals during the maintenance period. Statistical significance was assessed nonparametrically at the group level using a cluster-based permutation approach with a cluster-forming threshold of *p* < 0.05 (two-tailed) and a corrected significance level of *p* < 0.05 (two-tailed; Maris and Oostenveld, 2007). Condition labels were randomly permuted 1,000 times.

## Results

Participants performed a force production task using a hand dynamometer, where in each experimental trial they were required to match one of two possible force targets under different feedback conditions (Fig. 1). In the Total Feedback condition, participants were visually provided with online performance feedback throughout each 5 s trial. In the Early Feedback condition, feedback was presented for only the first 1.5 s. As illustrated in Figure 1*B*, participants were able to accurately maintain force during the total feedback condition, but force production decayed when only early feedback was provided.

To investigate the temporal dynamics of continuous, visually guided error correction, we conducted two streams of data analysis. First, in the behavioral analysis, we compared participant performance between the total feedback and early feedback conditions by applying spectral analysis. This allowed us to identify potential oscillatory patterns in force generation during action maintenance. Second, in the EEG–behavior analysis, we used cross-correlation to investigate the relationship between EEG (in both the time and frequency domains) and two behavioral measures: force production and the degree of error, defined as deviation from the target force. For the EEG data, we systematically examined effects across theta, alpha, and beta frequency bands and excluded delta band due to potential movement-related artifacts.

### Behavioral results: spectral analysis

We began by exploring the oscillatory structure of behavior in our force target conditions (40 or 55% of MVC). Results showed that force generation had a ∼6 Hz oscillatory component that emerged only when performance feedback was available (Fig. 2*A*). In the raw data, this ∼6 Hz effect emerges in the context of a strong fractal component structure, with power decreasing as a function of frequency. To isolate our effect from this overall pattern, we removed the fractal component of this signal (Fig. 2*B*; see Materials and Methods, Dynamometer spectral analysis).

The effect emerges in this cleaned plot between 5.27 and 7.42 Hz, with congruent peak frequencies in the two force conditions (40% = 6.25 Hz; 55% = 6.05 Hz). To statistically test this effect, we used linear mixed modeling with feedback presence as a predictor of dynamometer power in each frequency bin. We focused our analyses over the range between 2 and 20 Hz to evade both slow trends that could not clearly be associated with oscillatory patterns in the dynamometer signal, as well as faster oscillations that were not the target of our hypotheses. Results were cluster-corrected for multiple comparisons (see Materials and Methods). This analysis confirmed the presence of a significant effect of feedback over the interval between 5.27 and 7.42 Hz (cluster *p* < 0.001).

Our approach also revealed significant effects of feedback in the delta range (3.32–4.88 Hz). This effect is not associated with a distinct peak in the spectrum in any condition but rather with a low power in a narrow frequency range in both feedback conditions, making interpretation difficult. With this in mind, we have focused analysis on effects in the theta range, where strong peaks in oscillatory power emerge.

### EEG results

Our main goal was to explore the relationship between behavioral performance and EEG activity during the maintenance period, and we approached this in three ways. Since ERPs cannot be computed meaningfully in a fully continuous task, we focus on the cross-correlation between EEG activity and behavior. First, we examined the cross-correlation between overall behavioral performance and the broadband EEG signal, allowing us to identify general patterns of neural activity linked to task performance. Next, we focused on the relationship between behavioral error, defined as the absolute distance from the target, and EEG. This included both broadband EEG and power within the theta (4–8 Hz), alpha (8–12 Hz), and beta (12–30 Hz) frequency bands. Finally, we calculated the cross-correlation between the behavioral oscillatory pattern and power in the theta, alpha, and beta frequency bands described above.

#### Cross-correlation between performance and broadband EEG

For each trial, we computed the cross-correlation between behavioral exertion and EEG. The two signals were shifted relative to each other in time units of 0.01 s, to a maximum lag of 1 s, with correlation computed at each step.

**Figure 3.**
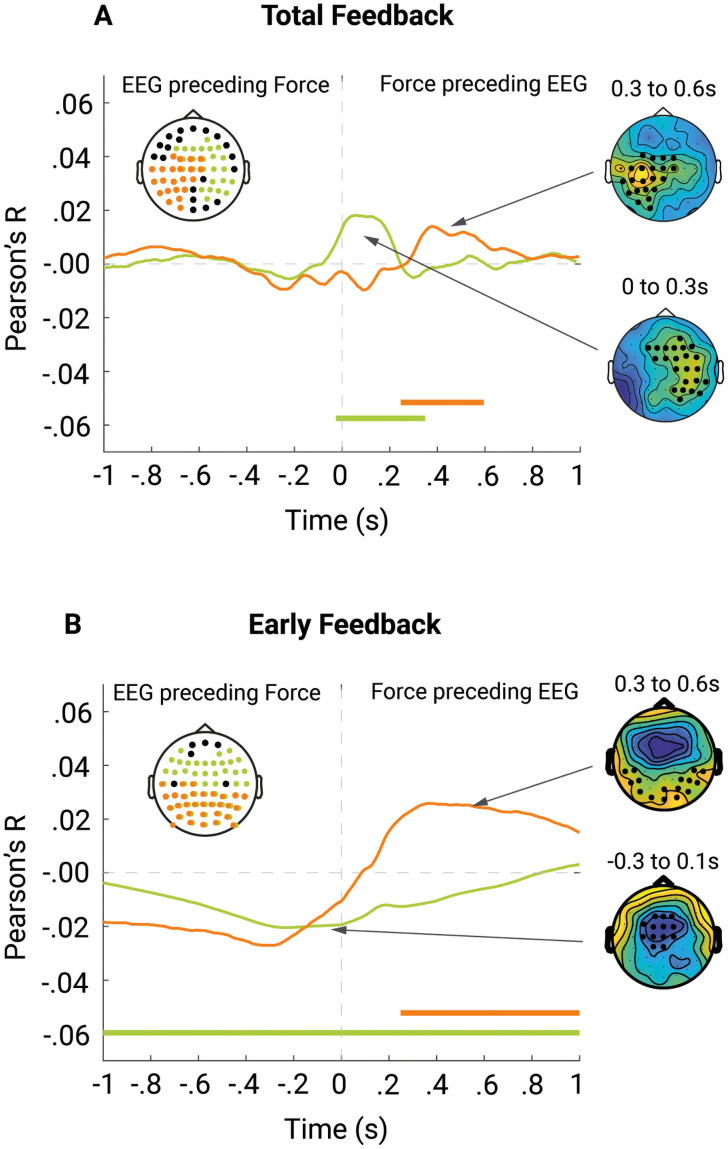
Cross-correlation between behavioral performance and broadband EEG signal in the Total Feedback (***A***) and Early Feedback (***B***) conditions. Positive and negative lags indicate whether EEG activity precedes or follows behavioral performance. Significant clusters are highlighted in green and orange, with the corresponding time intervals marked with the same color code at the bottom. The white topoplot identifies the sensors contributing to these significant clusters, while additional topographical plots illustrate the spatial distribution of EEG activity at key peaks within each condition and the relative significant channels highlighted.

In the total feedback condition, we found that the behavioral data were positively correlated with the EEG with significant clusters at positive time lags (−20 to 350 ms and 300–600 ms). This indicates a relationship in which dynamometer activity predicted the magnitude of the subsequent EEG signal. These effects emerge in EEG recorded from central electrodes, consistent with generators in the somatosensory cortex (Fig. 3*A*).

In the early feedback condition, we observed a prolonged negative correlation between the EEG signals and force exertion, spanning from positive to negative time lags across the entire scalp (Fig. 3*B*). This appears to reflect the greater decay in behavioral performance in this condition. Without continuous feedback, participants likely relied more on internal representations of the target force, which decayed over time. The widespread nature of the correlation suggests that, in the absence of external feedback, neural activity tracks a general decline in performance rather than discrete corrective adjustments. Additionally, we identified a positive correlation at positive time lags, specifically from 250 ms to 1 s, localized in the posterior region of the scalp. We interpret this as reflecting an evaluation of force exertion in the absence of online feedback, potentially supporting recalibration based on internal sensory predictions. Analyses revealed no main effect between the two force target (40 vs 55% MVC), and they showed no reliable differences in either the total feedback or the early feedback conditions.

#### Cross-correlation between behavioral error and broadband EEG

**Figure 4.**
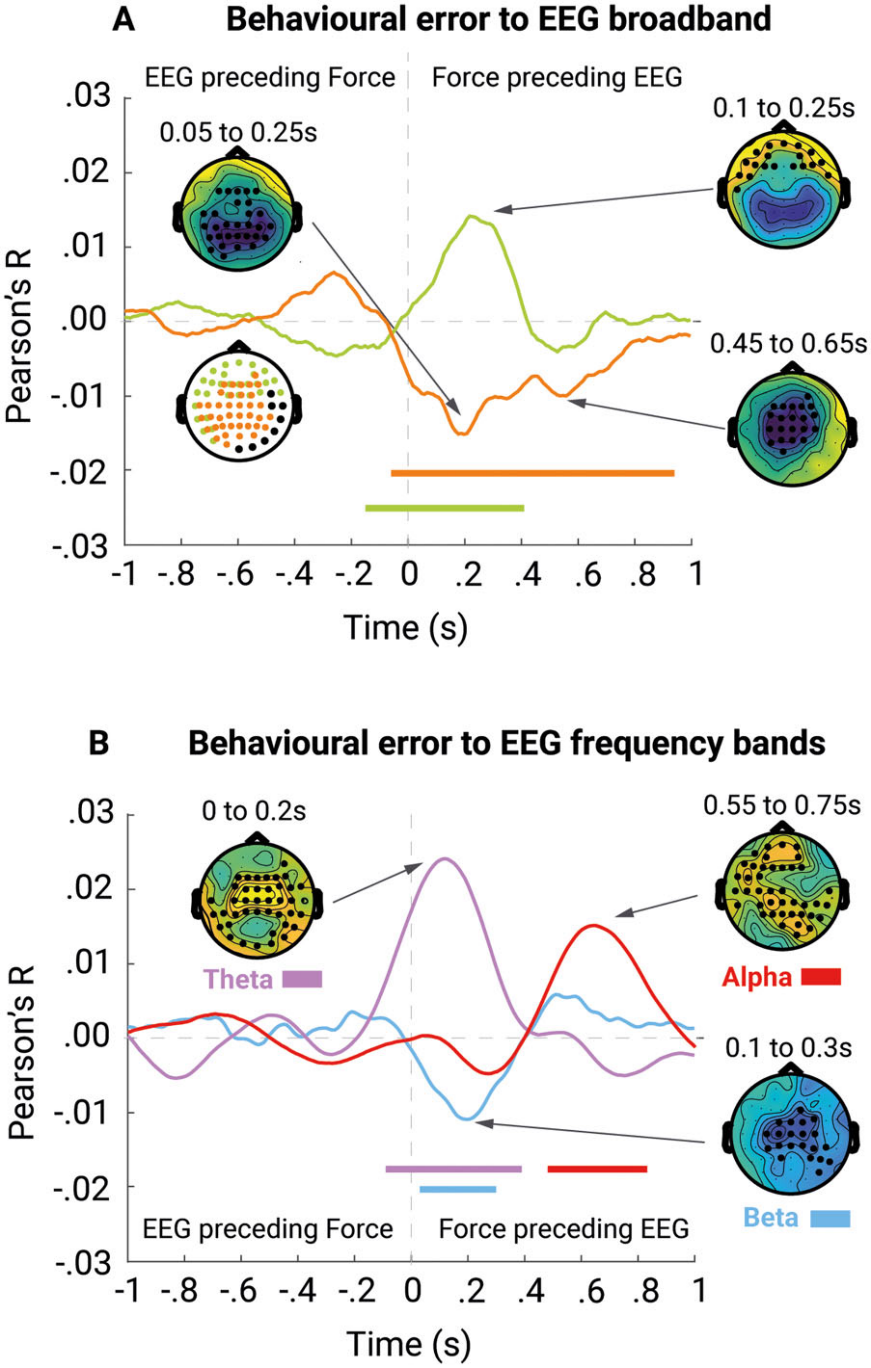
Cross-correlation between behavioral error from the target and broadband (***A***) or frequency band-specific (***B***) EEG signal in the Total Feedback condition. Positive and negative lags indicate whether EEG activity precedes or follows behavioral performance. Significant clusters are highlighted in different colors, with the corresponding time intervals marked with the same color code on top. The white topoplot identifies the sensors contributing to these significant clusters, while additional topographical plots illustrate the spatial distribution of EEG activity at key peaks within each condition, with significant channels highlighted.

When examining the relationship between EEG broadband signals and absolute error from the target, we restricted our analysis to the total feedback condition only, as performance in the early feedback condition consistently decayed over time, and error from the target no longer discretely reflected variance in motor control. For each total feedback trial, we computed the cross-correlation between behavioral error and EEG in 0.1 s steps with a maximum lag of 1 s. Two clusters emerged at early positive lags (Fig. 4*A*), reflecting a situation where dynamometer activity predicted the amplitude of the subsequent EEG signal. In the earlier cluster, we observed a positive correlation, while the later cluster was defined by negative correlation, such that a higher absolute distance from the target (i.e., error) was associated with reduced EEG amplitude. The negative cluster observed at −0.50 to 940 ms spanned from early posterior to central electrodes. The topography of this effect is consistent with generators in the medial prefrontal and anterior cingulate cortex, brain areas known to respond to error commission. The positive cluster at time lag *−*140 to 410 ms appears to reflect a generator in the frontal cortex. This may indicate anticipatory processes or preparatory adjustments preceding changes in behavioral output, potentially reflecting proactive monitoring and control mechanisms. No differences between the two force target (40 vs 55% MVC) were found in the total feedback condition.

#### Cross-correlation between behavioral error and EEG power in theta, alpha, and beta bands

We computed the cross-correlation between absolute behavioral error and EEG power in targeted spectra in the total feedback condition, allowing for a maximum lag of 1 s. EEG power was estimated using the Hilbert transform to obtain the instantaneous power in the theta (4–8 Hz), alpha (8–12 Hz), and beta (13–30 Hz) frequency bands. Our analysis revealed a significant positive cluster in each frequency band (with three independent analyses), all occurring at positive lags (Fig. 4*B*). This indicates that fluctuations in absolute behavioral error preceded corresponding changes in EEG power. The first significant cluster appeared in the theta band, suggesting an early neural response to behavioral error. Shortly thereafter, a significant cluster emerged in the beta band, overlapping temporally with theta but peaking slightly later. Finally, a significant alpha cluster emerged as the theta and beta effects began to decay. This temporal progression suggests a sequential recruitment of oscillatory activity, potentially reflecting distinct stages of error processing, motor correction, and feedback monitoring. No differences between the two force target (40 vs 55% MVC) were found in the total feedback condition.

#### Cross-correlation between behavioral oscillatory power and EEG power in theta, alpha, and beta bands

**Figure 5.**
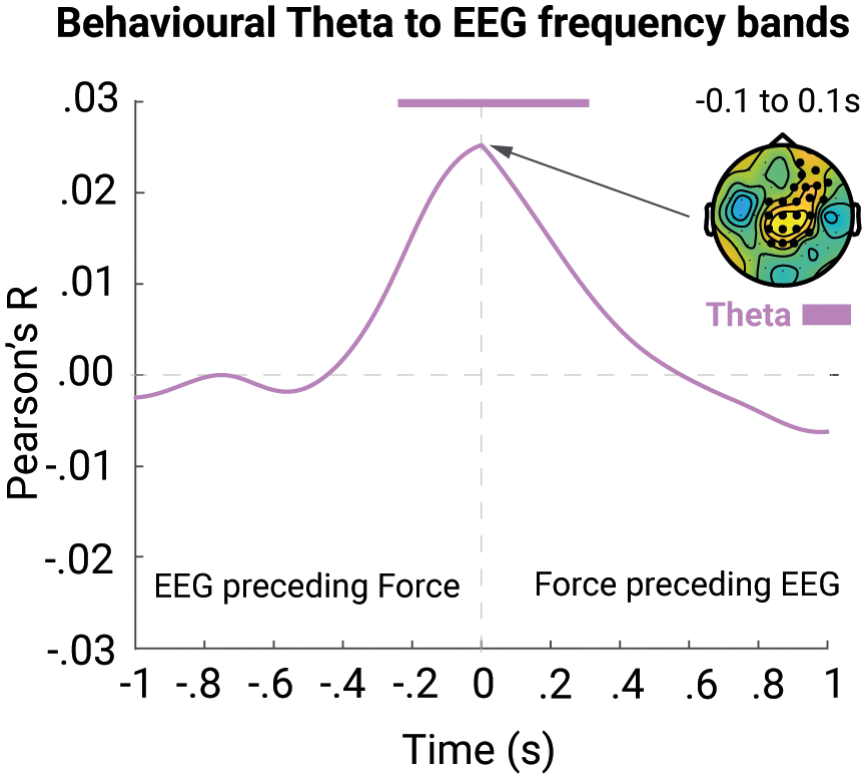
Cross-correlation between behavioral and EEG power in the theta band in the Total Feedback condition. Positive and negative lags indicate whether EEG activity precedes or follows behavioral performance. The time course of the significant cluster is presented, with the corresponding time intervals marked with the same color code on top. The topographical plots illustrate the spatial distribution of EEG activity at the key peak; significant channels are highlighted.

Finally, we examined the cross-correlation between the power of behavioral oscillations in the total feedback condition and EEG power, allowing for a maximum lag of 1 s. Behavioral oscillations were identified in the 5.5–7.2 Hz range, according to behavioral results, and their instantaneous power was again extracted using the Hilbert transform. Similarly, EEG power was identified in the theta (4–8 Hz), alpha (8–12 Hz), and beta (12–30 Hz) frequency bands. Our analysis revealed a single significant positive cluster in the theta frequency band, occurring in rough synchrony with changes in behavioral oscillation (Fig. 5). This indicates that fluctuations in the power of ∼6 Hz oscillations in force generation precede corresponding changes in theta power in the EEG. No significant clusters were observed in the alpha or beta bands. This result suggests a role of theta oscillations in processing force-related feedback. We additionally performed a complementary coherence analysis (Schoffelen et al., 2005) between EEG and behavioral theta activity (∼5.3–7.4 Hz), which yielded convergent sensor-level results (Supplementary Material Figs. 3, 5). A further peak-triggered ERP analysis, time-locked to behavioral theta-band force peaks, showed a consistent pattern, with source reconstruction (LCMV beamformer) suggesting a small parietal cluster (Supplementary Material Fig. 4).

## Discussion

We investigated the temporal dynamics of visually guided error correction during a task that required participants to continuously adjust hand force to match a target. Behaviorally, we found that continuous visual feedback facilitated error correction, helping participants maintain their force output close to the target. In the presence of feedback, performance exhibited a peak of performance fluctuation centered at ∼6 Hz, suggesting that correction was largely implemented at this rate.

The emergence of this specific frequency band is intriguing, as oscillations in this theta range have been observed across a variety of everyday behaviors requiring ongoing monitoring and fine adjustments, such as speech production, planning, typing, and visuomotor coordination (Vallbo and Wessberg, 1993; Yamaguchi et al., 2013; Kaplan et al., 2020; Ten Oever et al., 2024). This has led to the proposal that theta oscillations may serve as an internal metronome, facilitating the continuous evaluation and adjustment of motor output in response to performance errors (Cohen, 2014). Theta oscillations in behavior emerged in our results only in the total feedback condition. In the early feedback condition, where visual feedback was discontinued early in the trial, force output instead gradually decayed without evidence of correction (see also Vaillancourt et al., 2003; Limonta et al., 2015; Abolins and Latash, 2022).

One potential concern is that sustained high force exertion across many trials might have introduced cumulative fatigue. However, performance did not decline from early to late trials, and in some cases, it improved, providing no evidence for a progressive fatigue account (Supplementary Material Figs. 1, 2).

We examined the relationship between ongoing behavior and ongoing brain activity using a number of techniques. We began by examining the cross-correlation between force exertion and EEG amplitude as observed in the total feedback condition, finding a positive relationship in EEG electrodes located over the somatosensory cortex, suggesting a strong sensorimotor coupling during force adjustments. In contrast, in the early feedback condition, exertion was associated with activity in central and occipital channels. This pattern may reflect the gradual decline in performance over time: without ongoing visual feedback, neural activity appears to track the decay of force rather than actively signaling or correcting for errors.

Examining the correlation between broadband EEG activity and absolute error from the target, we found that larger errors were positively correlated with activity in frontal channels and negatively correlated with activity in central channels. The positive correlation in frontal regions may reflect increased engagement in performance monitoring, consistent with the role of the medial frontal cortex in monitoring and adjusting behavior (Luu et al., 2000; Ridderinkhof et al., 2004; Badre and Wagner, 2005). The negative correlation spanning from posterior to central regions may reflect engagement of medial frontal and central neural generators involved in error processing, which are well documented in the ERP literature (Trujillo and Allen, 2007; Wessel, 2012). Together, these effects suggest the involvement of partially distinct and complementary monitoring processes, with central activity tracking the error and frontal activity initiating the correction.

Next, we examined the cross-correlation between absolute error and EEG instantaneous power in the theta (4–8 Hz), alpha (8–12 Hz), and beta (12–30 Hz) frequency bands. Behavioral error was initially positively correlated with occipito-central theta, followed shortly by a negative correlation with central beta and, finally, a positive correlation with fronto-central alpha.

Although the peak correlation values are small and the resulting scalp maps appear spatially broad, this is to be expected in this EEG sensor data, where relationships between high-noise behavioral measures and neural activity typically produce small effect sizes and spatially smooth topographies. Nonetheless, the effects were consistent indicating that they reflect reliable, systematic relationships; future studies using MEG with MRI-guided source localization could more accurately identify the underlying cortical generators.

We interpret the early theta-band correlation as reflecting activity in medial frontal regions responsible for cognitive control and error processing (Nachev et al., 2008; Cavanagh and Frank, 2014; Cohen, 2014). The subsequent beta suppression is likely to reflect the initiation of motor responses in order to correct behavior and specifically to discontinue ongoing “status quo” performance (Engel and Fries, 2010). This interpretation is consistent with a recent study demonstrating a decrease in beta power during muscle contraction during reaching (Matta et al., 2025). Finally, subsequent frontal and occipital alpha modulation is consistent with the idea that participants adapt attentional control for visuomotor processing to monitor their performance adjustment (Rilk et al., 2011). This is in line with existing results suggesting connectivity in alpha and beta frequency bands underlies this kind of monitoring in visuomotor tasks (Palva and Palva, 2007; Hipp et al., 2011; Zhang et al., 2015). This temporal sequence suggests an error-processing loop, where errors first engage cognitive control (theta), followed by correction of motor activity (beta) and, finally, a recalibration of attentional and motor networks (alpha) to optimize subsequent performance. We do not interpret these frequency-specific effects as competing or directly comparable signals. Rather, we believe them to be parallel observations, with each frequency band reflecting a different functional component of continuous error processing with distinct temporal dynamics and spatial profile.

Recent work has emphasized the importance of transient, burst-like dynamics in motor control, particularly in the beta band. Studies examining beta-burst coupling with EMG and behavior have shown that brief, time-locked neural events can track motor output and error processing at the single-trial level (Wessel, 2020; Lundqvist et al., 2024; Graef et al., 2025). These approaches provide a valuable perspective by characterizing the fine temporal structure of neural responses during action. In contrast, the present study adopts a different but complementary perspective by focusing on the continuous coupling between ongoing behavior and ongoing neural activity over time. Together, these perspectives converge on the idea that motor control emerges from temporally structured interactions between neural activity and behavior, but they do so at different descriptive levels.

Finally, we cross-correlated the instantaneous power of the behavioral exertion signal in the significant frequency band with the instantaneous power of EEG activity in the theta, alpha, and beta frequency bands. We found a significant correlation with central theta, coherent with a role for theta oscillations in sensorimotor coordination (Cohen, 2016). This finding indicates that fluctuations in exertion are dynamically linked to neural processes involved in continuous feedback processing and error correction mechanisms. We further complemented this result with a coherence analysis between EEG and behavioral theta activity, which yielded convergent sensor-level effects, as well as a peak-triggered analysis time-locked to behavioral theta peaks, which showed a consistent pattern of activity. Source-level estimates from this analysis suggested a parietal distribution, which may reflect a convergence of visual and motor information during continuous performance monitoring, although this localization should be interpreted with caution given the constraints of EEG source reconstruction, particularly in the absence of subject-specific structural anatomy, for example, from MRI.

Together, these findings are consistent with a closed-loop sensorimotor process linking visual feedback and motor adjustment. However, the present data do not fully dissociate attentional resource allocation and coordinated visual–motor interactions, which remain important questions for future work.

In summary, our findings reveal the neural dynamics behind visually guided force control and error correction. Feedback processing involved a dynamic interplay of oscillatory activity, with theta, gamma, and alpha bands reflecting different stages of performance monitoring and motor adaptation. Together, these results provide insight into the neural dynamics of motor control and suggest that theta oscillations may serve as a bridge between sensory feedback and motor execution, facilitating adaptive behavior during completion of ongoing, continuous tasks.
